# Classifying breast cancer surgery: a novel, complexity-based system for oncological, oncoplastic and reconstructive procedures, and proof of principle by analysis of 1225 operations in 1166 patients

**DOI:** 10.1186/1471-2407-9-108

**Published:** 2009-04-08

**Authors:** Jürgen Hoffmann, Diethelm Wallwiener

**Affiliations:** 1 Department of Obstetrics and Gynaecology, University of Tübingen, Calwerstraße 7, 72076 Tübingen, Germany

## Abstract

**Background:**

One of the basic prerequisites for generating evidence-based data is the availability of classification systems. Attempts to date to classify breast cancer operations have focussed on specific problems, e.g. the avoidance of secondary corrective surgery for surgical defects, rather than taking a generic approach.

**Methods:**

Starting from an existing, simpler empirical scheme based on the complexity of breast surgical procedures, which was used in-house primarily in operative report-writing, a novel classification of ablative and breast-conserving procedures initially needed to be developed and elaborated systematically. To obtain proof of principle, a prospectively planned analysis of patient records for all major breast cancer-related operations performed at our breast centre in 2005 and 2006 was conducted using the new classification. Data were analysed using basic descriptive statistics such as frequency tables.

**Results:**

A novel two-type, six-tier classification system comprising 12 main categories, 13 subcategories and 39 sub-subcategories of oncological, oncoplastic and reconstructive breast cancer-related surgery was successfully developed. Our system permitted unequivocal classification, without exception, of all 1225 procedures performed in 1166 breast cancer patients in 2005 and 2006.

**Conclusion:**

Breast cancer-related surgical procedures can be generically classified according to their surgical complexity. Analysis of all major procedures performed at our breast centre during the study period provides proof of principle for this novel classification system. We envisage various applications for this classification, including uses in randomised clinical trials, guideline development, specialist surgical training, continuing professional development as well as quality of care and public health research.

## Background

The treatment of breast cancer is multimodal and generally involves surgery, radiation and neoadjuvant and/or adjuvant systemic therapy. Even today, these three basic approaches to breast cancer therapy differ considerably with regard to the availability of evidence-based medical data required to evaluate the effectiveness and quality of treatment [1,2]. Whereas systemic treatment of breast cancer and other malignancies has had a long tradition of clinical trials [3-5], generating a large body of data with high levels of evidence as defined by the Centre for Evidence-Based Medicine, Oxford, UK [6,7], the amount of evidence-based data available on radiation therapy is more limited [8,9]. As regards breast cancer surgery, evidence-based data are available practically only from studies comparing lumpectomy or quadrantectomy versus mastectomy, or, in more abstract terms, breast-conserving surgery (BCS) versus ablative breast surgery (ABS) [10-12].

Currently, systematically generated data are lacking for oncological (as opposed to plastic) breast surgery as well as for what has come to be known as "oncoplastic" surgery, i.e. surgery that combines both oncological and plastic surgery procedures in a single operation. Hence, it has not been possible to achieve for breast cancer surgery, particularly oncoplastic surgery, the high levels of evidence demanded by the advocates of quality-assured treatment and others [13-15]. In our opinion, this lack of evidence-based data in breast cancer surgery largely results from the absence of standardisation and classification of the vast number of different surgical procedures and techniques in use. We consider this to be especially true in the case of oncoplastic breast surgery [14,16,17].

So far, oncoplastic procedures have only been classed post-hoc with a view to anticipating future iatrogenic defects in order to obviate the need for their repair and minimise the cost and effort this involves [18,19]. To our knowledge, no classification of breast cancer surgical procedures has yet been devised, or published, on the basis of purely surgical criteria. However, such a classification system is needed if data are to be generated that will enable us to introduce the principles of evidence-based medicine into breast cancer surgery and assess our surgical methods in clinical trials to achieve high levels of evidence.

In the present paper, we propose the first comprehensive classification system capable of accommodating, on the basis of surgical complexity, any major oncological, oncoplastic or reconstructive procedure used in the surgical treatment of primary and locally recurrent breast cancer. As a proof of principle, we have reviewed the operative reports and hospital records of all female breast cancer patients who underwent oncological, oncoplastic or reconstructive surgical procedures in our department in 2005 and 2006, with the aim of assigning each operation to one specific category within our classification system.

## Methods

### Study objective, design and setting

The main objectives of our study were twofold: (1) to elaborate and refine an empirical scheme developed in-house for classifying major oncological, oncoplastic and reconstructive surgical procedures commonly performed in breast cancer surgery; and (2) to demonstrate the general applicability of our novel classification to all breast cancer procedures by carrying out a proof-of-principle analysis of all major breast cancer operations performed at the Tübingen University Breast Centre in 2005 and 2006.

This study was designed as a prospective analysis of existing hospital treatment records. It was noninterventional, and no patient-identifiable data were used in the analysis. According to the relevant German laws and regulations, the study therefore did not require approval by an independent ethics committee or institutional review board, or informed patient consent. This was confirmed in writing by the Ethics Committee of the Medical Faculty of the University of Tübingen.

All breast surgical procedures were performed for the treatment of any stage of breast cancer at the Department of Obstetrics and Gynaecology, University of Tübingen during the study period from 1 January 2005 to 31 December 2006.

### Definitions

#### Oncoplastic surgery

Whereas "oncological surgery" and "reconstructive surgery" are well-established terms requiring no further definition in the present context, "oncoplastic surgery" is a relatively new concept for which there is no established, generally accepted definition [20]. For the purposes of our classification studies, we used the term "oncoplastic surgery" to refer to any surgical procedure in which the primary surgical treatment strategy involved plastic surgical techniques for partial or complete reconstruction of the breast or for correction of surgical defects to the thoracic wall. This definition was based on the concept of "tumour-specific immediate reconstruction" (TSIR) proposed by Audretsch et al. [21] and Bostwick and collaborators [22]. In a broader sense, the terms "oncoplastic surgery" and TSIR both also encompass the extension of breast-conserving surgery to include the concomitant application of plastic surgical techniques, immediate reconstruction in the context of skin-sparing or modified radical mastectomies and thoracic wall reconstruction after radical local surgery. Ultimately, both terms include all procedures that are used to avoid defects or integrate defect correction into the primary oncological procedure and that are performed under the same oncological safety criteria as the conventional surgical procedures.

#### Major surgical procedure

This term was used to describe any immediate or delayed operation that involved a change of classification category, e.g. conversion from a breast-conserving procedure to mastectomy or from mastectomy to defect repair with flap techniques in local radical excision. Secondary operations such as re-excision of breast tissue due to involved margins were not considered major procedures provided they did not require reclassification.

#### Complexity

This was used to refer to the technical difficulty of surgical procedures.

### Classification system

An initially simpler scheme used in-house to class breast cancer-related operations was originally developed in our department as a means of facilitating administrative tasks, especially the efficient and systematic preparation of operative reports. This rudimentary system was considered a suitable starting point for a comprehensive, systematic classification system based on the surgical complexity of breast cancer procedures.

### Patients

#### Inclusion criteria

All women who underwent surgery for breast cancer at the Breast Centre of the University of Tübingen during the period from 1 January 2005 to 31 December 2006 were eligible for inclusion in the study.

#### Exclusion criteria

There were no exclusion criteria. All female breast cancer patients receiving major oncological, oncoplastic or reconstructive surgery at the Breast Centre of the University of Tübingen during the study period were to be included in the analysis.

### Data analysis

#### Patient data

Patients' operative reports were reviewed by the investigators with regard to type of surgical procedure or procedures performed and the extent and complexity of surgery. Surgical procedures were assigned to one of sub-subcategories, subcategories or main categories of the proposed surgical classification system. Any procedure that could not be unequivocally assigned to a category was to be considered nonclassifiable.

#### Statistical methods

Due to the nature of the study, statistical analysis was limited to basic descriptive methods. Essentially, frequency tables were generated and percentages calculated using Microsoft Excel^® ^2002 (Microsoft Corporation, Redmond, WA, USA).

## Results

### The novel classification system

**Figure 1:**
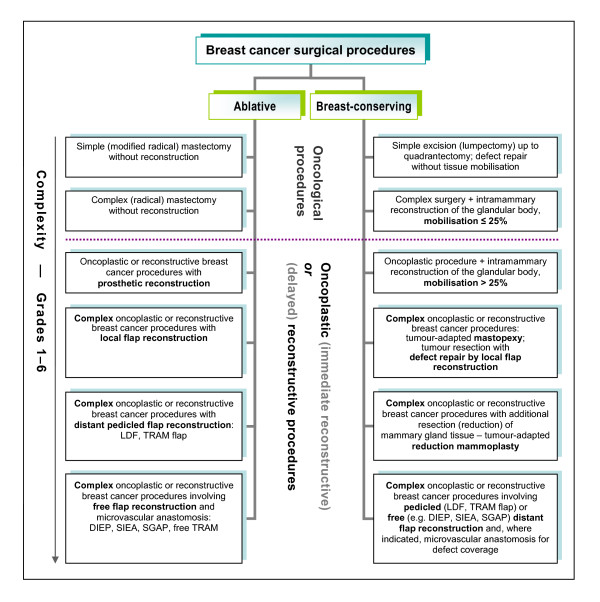
**Classification of ablative and breast-conserving surgical procedures for the treatment of breast cancer**. The system is based on six levels of complexity of oncological, oncoplastic and delayed reconstructive surgery (simplified representation).

The pre-existing rudimentary classification used in our breast centre to facilitate administrative tasks such as preparing operative reports was successfully expanded, refined and placed on a systematic basis that reflected the complexity of the procedures. A schematic representation of the resulting novel, complexity-based systematic classification system of breast cancer-related surgical procedures is given in Figure 1, which shows the two-type, six-tier basic structure of the classification. A complete and detailed listing of the 12 main categories and all sub- and sub-subcategories with their definitions is given in Table 1.

**Table 1:** Complexity-based classification of oncological, oncoplastic and delayed reconstructive procedures in ablative (A) and breast-conserving (B) breast cancer-related surgery.

A. Ablative surgical procedures	
**1**.	**Simple ablative breast cancer surgery**
	Modified radical mastectomy or excision of a local recurrence after ablation (without reconstruction)

**2**.	**Complex ablative breast cancer surgery**
	Radical mastectomy or excision of a local recurrence after ablation (with removal of pectoral muscles and without reconstruction)

**3**.	**Oncoplastic ablative surgical procedures for breast cancer treatment with prosthetic reconstruction, or reconstruction after ablation**
***a*.**	***Modified radical mastectomy with immediate prosthetic reconstruction***
*1*.	*Modified radical mastectomy with immediate placement of a tissue expander*
*2*.	*Modified radical mastectomy with immediate placement of a permanent implant*
***b*.**	***Skin-sparing mastectomy with immediate prosthetic reconstruction***
*1*.	*Skin-sparing mastectomy (SSM) and immediate placement of an implant*
*2*.	*Nipple-sparing mastectomy (NSM) and immediate placement of an implant*
***c*.**	***Delayed prosthetic reconstruction after mastectomy***
*1*.	*Delayed reconstruction after mastectomy: placement of a tissue expander*
*2*.	*Delayed reconstruction after mastectomy: placement of a permanent implant*
***d*.**	***Implant-related procedures after implant reconstruction***
*1*.	*Tissue expander removal and placement of a permanent implant*
*2*.	*Implant exchange and breast remodelling in capsular contracture*

**4**.	**Complex oncoplastic ablative breast cancer surgery involving defect repair with local flaps or free skin grafts (also in extensive chest wall recurrence)**
*1*.	*Transposition flaps (e.g. the thoracoepigastric flap)*
*2*.	*Rotation flaps*
*3*.	*Local advancement flaps*
*4*.	*Free skin transplants (e.g. a mesh graft)*

**5**.	**Complex oncoplastic ablative breast cancer surgery with reconstruction or defect repair using distant pedicled flaps (also in extensive chest wall recurrence)**
***a*.**	***Conventional latissimus dorsi (LD) flaps (surgical or endoscopic harvest)***
*1*.	*Conventional LD flap without or with an implant for immediate breast reconstruction as part of ablative surgery (modified radical mastectomy, SSM, NSM)*
*2*.	*Conventional LD flap (usually without an implant) for repair of chest wall defects*
*3*.	*Conventional LD flap without or with an implant for delayed breast reconstruction after ablative surgery*
*4*.	*Conventional LD flap without or with an implant for autologous reconstruction or combined autologous and alloplastic reconstruction in prosthesis-related complications*
***b*.**	***Extended latissimus dorsi (LD) flaps***
*1*.	*Extended LD flap for immediate breast reconstruction as part of ablative surgery (modified radical mastectomy, SSM, NSM)*
*2*.	*Extended LD flap for repair of chest wall defects*
*3*.	*Extended LD flap for delayed breast reconstruction after ablative surgery*
*4*.	*Extended LD flap for conversion to autologous reconstruction in prosthesis-related complications*
***c*.**	***Transverse rectus abdominis (TRAM) flaps***
*1*.	*TRAM flap for immediate breast reconstruction as part of ablative surgery (modified radical mastectomy, SSM, NSM)*
*2*.	*TRAM flap for repair of chest wall defects*
*3*.	*TRAM flap for delayed breast reconstruction after ablative surgery*
*4*.	*TRAM flap for conversion to autologous reconstruction in prosthesis-related complications*

**6**.	**Complex oncoplastic ablative breast cancer surgery involving reconstruction or defect repair using free flaps with microvascular anastomosis (e.g. DIEP, SIEA, SGAP or free TRAM flaps) (also in extensive chest wall recurrence)**
*1*.	*Free flap for immediate breast reconstruction as part of ablative surgery (modified radical mastectomy, SSM, NSM)*
*2*.	*Free flap for repair of chest wall defects*
*3*.	*Free flap for delayed breast reconstruction after ablative surgery*
*4*.	*Free flap for conversion to autologous reconstruction in prosthesis-related complications*

**B. Breast-conserving surgical procedures**	

**1**.	**Simple breast-conserving cancer surgery**
	(histologically complete tumour excision, performed as a "wide excision" up to quadrantectomy with defect repair involving only direct apposition without mobilisation of glandular tissue or skin flaps)

**2**.	**Complex breast-conserving cancer surgery**
	(additional intramammary reconstruction of the mammary gland by mobilisation of subcutaneous or epifascial glandular lobes and, if necessary, mobilisation of the skin envelope for defect repair of ≤ 25% of the area of the breast)

**3**.	**Oncoplastic breast-conserving surgery**
	(additional intramammary reconstruction of the mammary gland by mobilisation of subcutaneous or epifascial glandular lobes and, if necessary, mobilisation of the skin envelope for defect repair of > 25% of the area of the breast)

**4**.	**Complex oncoplastic breast-conserving surgery**
***a*.**	***Tumour-adapted mastopexy (breast lift) without additional removal of healthy breast tissue***
	(selection of skin incision pattern depending on amount of excess skin and desired scar pattern, i.e. purely circumareolar, vertical, segmental or "inverted T")
*1*.	*Central pedicles*
*2*.	*Inferior-central pedicles*
*3*.	*Cranial pedicles*
*4*.	*Modified B mammoplasty*
*5*.	*Free nipple transfer*
***b*.**	***Tumour resection with defect repair using local flaps without or with skin replacement***
*1*.	*Transposition flaps (e.g. the thoracoepigastric flap)*
*2*.	*Rotation flaps*

**5**.	**Complex oncoplastic breast-conserving surgery with additional resection (reduction) of mammary gland tissue – Tumour-adapted reduction mammoplasty**
	(selection of skin incision pattern depending on amount of excess skin and desired scar pattern, i.e. purely circumareolar, vertical, segmental or "inverted T")
*1*.	*Central pedicles*
*2*.	*Inferior-central pedicles*
*3*.	*Cranial pedicles*
*4*.	*Free nipple transfer*

**6**.	**Complex oncoplastic breast-conserving surgery involving defect repair using distant pedicled flaps**
***a*.**	***Tumour resection with partial volume replacement using an endoscopically harvested latissimus dorsi flap***
***b*.**	***Tumour and skin resection with partial volume and skin replacement using a latissimus dorsi flap with a skin island***
***c*.**	***Tumour and, if required, skin resection with partial volume replacement and, if indicated, skin replacement using a pedicled TRAM flap***
***d*.**	***Tumour excision and, if indicated, skin resection with partial volume replacement and, if indicated, skin replacement using free flaps with microvascular anastomosis (e.g. DIEP, SIEA or SGAP flaps)***

### Patients and operations

In total, 1166 women with breast cancer underwent 1225 major oncological, oncoplastic or reconstructive surgical procedures at our Breast Centre between 1 January 2005 and 31 December 2006. All patients and procedures were included in the present analysis.

### Proof-of-principle analysis

The results of the analysis based on our classification system are summarised in Table 2. Most importantly, our novel classification system successfully accommodated all 1225 major breast cancer procedures performed at our Breast Centre during the two-year study period, leaving no unclassifiable procedures.

**Table 2:** Classification-based analysis of all 1225 major procedures in 1166 breast cancer patients in 2005 and 2006

			**ABS**	**BCS**
				
**1**			231 (18.9%*)	9 (0.7%*)
		
**2**			**9 (0.7%)**	**186 (15.2%)**
		
**3**			**206 (16.8%)**	**202 (16.5%)**
		
	3.a		25	--
			
		*1*	*24*	--
		*2*	*1*	--
			
	3.b		74	--
			
		*1*	*66*	--
		*2*	*8*	--
			
	3.c		13	--
			
		*1*	*13*	--
		*2*	*0*	--
			
	3.d		94	--
			
		*1*	*67*	--
		*2*	*27*	--
		
**4**			**27 (2.2%)**	**152 (12.4%)**
		
	4.a		--	144
			
		*1*	*8*	*48*
		*2*	*3*	*11*
		*3*	*16*	*3*
		*4*	*0*	*82*
		*5*	--	*0*
			
	4.b		--	8
			
		*1*	--	*7*
		*2*	--	*1*
		
**5**			**75 (6.1%)**	**123 (10.0%)**
		
	5.a		2	--
			
		*1*	*0*	*14*
		*2*	*2*	*85*
		*3*	*0*	*21*
		*4*	*0*	*3*
			
	5.b		71	--
			
		*1*	*60*	--
		*2*	*0*	--
		*3*	*7*	--
		*4*	*4*	--
			
	5.c		2	--
			
		*1*	*0*	--
		*2*	*0*	--
		*3*	*1*	--
		*4*	*1*	--
		
**6**			**4 (0.3%)**	**1 (0.0%)**
		
	6.a		--	1
		*1*	*1*	--
		*2*	*0*	--
		*3*	*1*	--
		*4*	*2*	--
			
	6.b		--	0
			
	6.c		--	0
			
	6.d		--	0
		
**Total of main categories 1–6**			**552 (45.1%)**	**673 (54.9%)**

All patients underwent ablative breast surgery (ABS) or breast-conserving surgery (BCS) at the Breast Centre, Department of Obstetrics and Gynaecology, Tübingen University Hospital.

* % of 1225 procedures

-- nonexistent category

As a secondary result of our proof-of-principle analysis, we obtained a remarkably detailed complexity profile of the breast cancer-related surgery performed at our Breast Centre during the study period (Table 2). Inspection of the main-category data alone already gives a reasonably accurate picture of the breast cancer surgery performed, showing that 45% (552) of all 1225 major procedures were ablative (ABS) and 55% (673) were breast-conserving (BCS) operations. More specifically, about 19% (of 1225 procedures) were simple ablative (Category A.1, see Table 1) and about 15% were oncoplastic ablative procedures (Categories A.3.a+b; A.4; A.5.a.1+2; A.5.b.1+2; A.5.c.1+2; A.6.1+2), thus accounting for the vast majority of ablative procedures. As regards breast-conserving surgery, complex BCS (Category B.2; 15%), oncoplastic BCS (B.3; 17%), complex oncoplastic BCS (B.4; 12%) and complex oncoplastic BCS with additional resection of mammary gland tissue (B.5; 10%) accounted for all but 10 BCS procedures performed at the Tübingen University Breast Centre in 2005 and 2006.

## Discussion

### Classification system

The complexity-based classification of breast surgical procedures we propose here is, to the best of our knowledge, the first of its kind. No other generally applicable system for the classification of breast cancer-related surgery appears to have been published in the literature. Classifications developed to date have been based on post-therapeutic (i.e. after surgery and radiation treatment) analysis of defects resulting from breast-conserving treatment and the secondary repair surgery they entail [18,19]. Based on such approaches, Munhoz and colleagues [23] very recently published a classification system of breast-conserving surgery-related defects which facilitates the prospective identification of individual, patient-specific surgical solutions by using an algorithm for selecting the appropriate surgical technique. However, all of these approaches are limited to the specific purpose for which they were developed and are not suitable for a wider range of more general applications, e.g. randomised controlled trials (RCTs) to generate evidence-based breast surgery data, which are currently still lacking [20,24].

The selection of an appropriate, oncologically safe surgical procedure depends on numerous factors relating to, inter alia, the individual patient, tumour characteristics and surgeon's skills. In practice, it is not unusual that evaluation of a patient will result in her being offered several surgical treatment options, frequently of different complexity. We decided to base our classification on technical sophistication and complexity of surgical procedures because this principle of classification is independent of complex factors such as the individual patients' treatment needs or surgeons' preferences, thus rendering our system more practical and suitable for the purposes of scientific research.

We consider the advantages of our novel classification system to be that it is clinically orientated, easy to apply and sufficiently differentiated without being overloaded.

Based on our experience of 1225 major breast cancer procedures in 1166 patients, we are confident that the complexity-based classification system we propose here is sufficiently comprehensive to accommodate virtually any breast cancer-related surgical procedure. Our classification is not limited to the procedures we actually performed but rather also accommodates other types of procedures we have not yet performed ourselves or for which we saw no compelling indication in any of our patients.

### Proof of principle

All 1225 major breast cancer-related surgical procedures performed at the Tübingen University Breast Centre during the 2005–2006 study period were assignable to exactly one category within our complexity-based classification system, thus providing proof of principle for this novel approach.

### Potential applications and outlook

We can envisage numerous potential applications for our classification system in various settings. For instance, in an everyday clinical setting, it can be used to produce operative reports more systematically and efficiently. At an institutional level, our classification offers breast centres a simple and practical means of obtaining a transparent profile and overview of the range of breast cancer surgical procedures they offer by determining the frequencies of operations of the various classification categories as percentages of total caseload. The proof-of-principle analysis shown in Table 2 is an example of such a frequency analysis. Over longer periods of time, such analyses could identify and illustrate any changes and trends in a hospital's capabilities and the range of operations it offers. At the level of the individual breast cancer surgeon, the classification may also lend itself to categorising oncological, oncoplastic and reconstructive surgical skills. Conversely, higher surgical training curricula and continuing professional development (CPD) programmes for breast cancer surgeons could be based on the classification system we propose.

Furthermore, at the supra-institutional level, our classification could be used for inter-hospital comparisons or even in the context of benchmarking systems to achieve greater transparency as regards the current situation and quality of care in the surgical management of breast cancer. Similar comparisons at the national and even international levels are also conceivable.

Lastly, we believe that the novel classification system we propose will make it easier to address scientific questions and issues in areas such as quality of care and public health, and may enable a more scientific approach to breast cancer surgery as such. As a result, data generated from such research could ultimately contribute to the development of evidence-based guidelines for the surgical treatment of breast cancer.

## Conclusion

Breast cancer-related surgical procedures can be generically classified according to their surgical complexity. Proof of principle for our novel classification system was obtained by descriptive statistical analysis which demonstrated that our classification enabled unequivocal representation all 1225 major procedures performed in 1166 women with breast cancer at the Tübingen University Breast Centre during the study period from 1 January 2005 to 31 December 2006. Our classification system is simple and easily applicable, sufficiently comprehensive and differentiated without being too detailed or intricate. We therefore consider it to be a suitable tool for a number of purposes in various areas, including randomised clinical trials and evidence-based data generation, guideline development, specialist surgical training, continuing professional development, as well as quality of care and public health research relating to breast cancer surgery.

## Competing interests

The authors declare that they have no competing interests.

## Authors' contributions

JH conceived of the original scheme from which the classification was developed, participated in the study design, collected and analysed the data, drafted the manuscript and finalised it. DW conceived of the proof-of-principle study, participated in the elaboration of the classification system, conceived of the range of its applications and revised the manuscript. Both authors read and approved the final manuscript.

## Pre-publication history

The pre-publication history for this paper can be accessed here:

http://www.biomedcentral.com/1471-2407/9/108/prepub
